# Adrenal Solitary Fibrous Tumor: A Case Report

**DOI:** 10.7759/cureus.50300

**Published:** 2023-12-10

**Authors:** Elena Casademunt-Gras, Isabel Salinas, Pau Moreno Santabarbara, Gustavo Tapia Melendo, Jordi L Reverter

**Affiliations:** 1 Endocrinology and Nutrition, Hospital Universitari Germans Trias i Pujol, Barcelona, ESP; 2 General and Digestive Surgery, Hospital Universitari Germans Trias i Pujol, Barcelona, ESP; 3 Clinical Pathology, Hospital Universitari Germans Trias i Pujol, Barcelona, ESP

**Keywords:** mesenchymal neoplasm, adrenalectomy, stat6, adrenal gland, solitary fibrous tumor

## Abstract

Solitary fibrous tumor is a mesenchymal neoplasm that first appeared in the literature as a pleural lesion, but over the last decades, it has been reported in many extrathoracic sites. Primary solitary fibrous tumor in the adrenal gland is very uncommon. Its biological behavior is variable but mostly benign.

We report here a case of an apparently healthy woman who, in the context of the study of limb paresthesias, was diagnosed with an adrenal incidentaloma. Laboratory tests were performed, and no hormone hyperfunction was detected. Subsequently, a right adrenalectomy was performed, and the pathological study confirmed a solitary fibrous tumor. To the best of our knowledge, this is the 19th case reported in the literature of a primary solitary fibrous tumor originating from the adrenal gland and, notably, the first documented instance in Spain.

## Introduction

Solitary fibrous tumor (SFT) comprises a histologic spectrum of rarely metastasizing fibroblastic mesenchymal neoplasms that originate in the pleura. Extra-pleural sites have been described, including the skin [1], peritoneum, genitourinary system [2], and pelvis. They are mostly benign neoplasms composed of predominantly fibrous lesions containing large collagenized areas and hyalinized vessels. Nevertheless, a comprehensive work-up should exclude malignant lesions, especially focusing on radiologic and histopathological features of the tumor. 

Solitary fibrous adrenal tumor (SFAT) is an extremely rare mesenchymal neoplasm, with only a few cases reported in the literature. Ambardjieva et al. conducted a review of published data up to 2021 and identified 12 reported cases of SFAT [3]. The patients' ages ranged from 23 to 77 years, and SFAT was often discovered incidentally during imaging intended for other reasons, without symptoms or lumbar pain. SFAT was predominantly found in an isolated form, occurring on both the left and right adrenal glands (bilateral presentation was observed in only one case). The median diameter of the tumors was 9.85 cm, with a range of 2.5 to 18 cm. 

Surgical resection emerged as the primary treatment approach, with no adjuvant therapy administered. Follow-up data, spanning from seven to 23 months, were available for seven patients. All patients were alive without any recurrence of disease or development of metastasis. The objective of this case report is to introduce the first instance of SFAT documented in Spain.

## Case presentation

**Figure 1:**
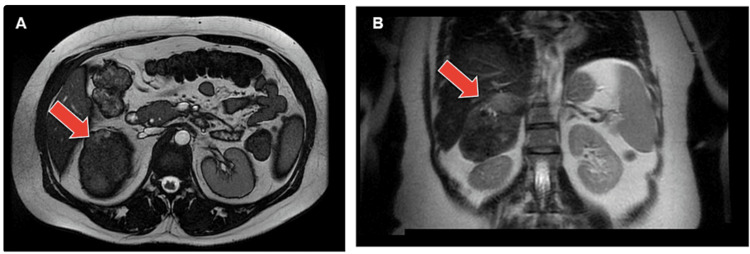
A and B - magnetic resonance of solitary fibrous tumor in the right adrenal gland

We present a clinical case involving a healthy 48-year-old woman who presented complaints of paresthesias and muscular weakness in her arms and legs. Cervical magnetic resonance imaging (MRI) revealed a disc bulge located within the C5-C6 vertebrae, resulting in moderate compression of the spinal cord. Additionally, posterior disc bulging was observed at the L4-L5 level. Notably, abdominal imaging highlighted a substantial mass positioned between the upper pole of the right kidney and the right hepatic lobe in the adrenal region, exhibiting a maximal diameter of 10 cm. The mass displayed a heterogeneous signal pattern, predominantly hypointense on T2-weighted sequences. Hyperintense regions were evident, and even a few minor cystic components were noticeable in its uppermost section. Following the intravenous administration of a gadolinium-based contrast agent, the mass exhibited progressive and heterogeneous enhancement. Restricted diffusion areas were identified within the mass. However, no T1 signal drop was observed, suggesting a potential fatty component. The mass exhibited well-defined margins. The left adrenal gland had normal dimensions and morphology. Given the observed features (Figure 1), it was imperative to consider the exclusion of adrenal malignancies such as adrenal carcinoma and pheochromocytoma.

**Figure 2:**
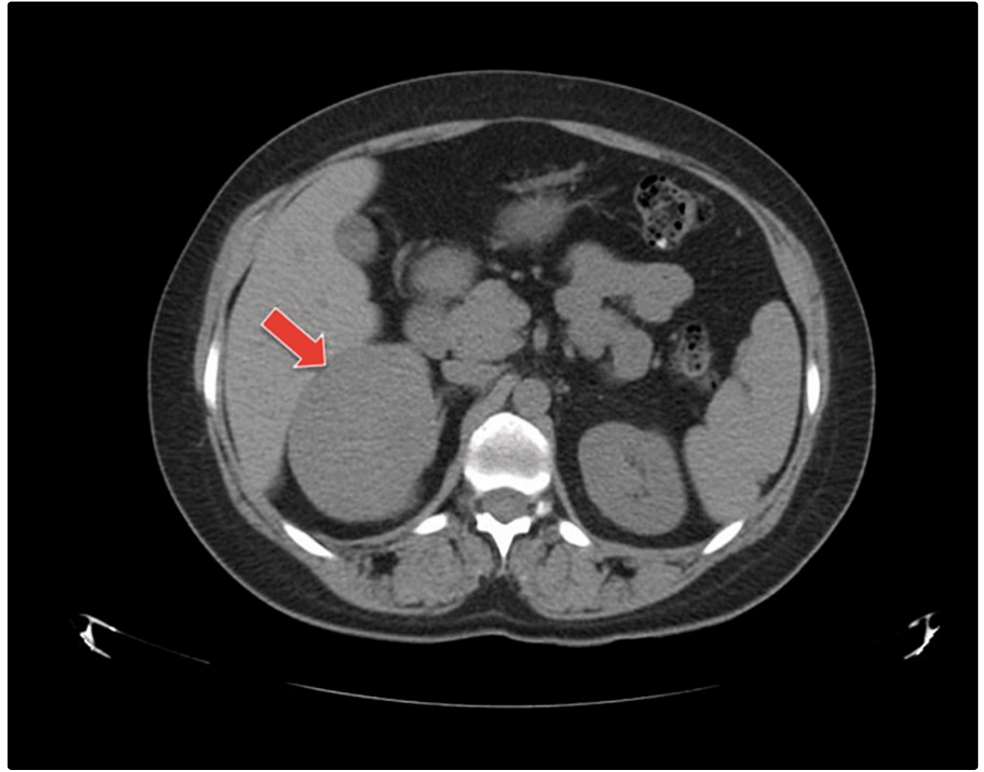
Computer tomography scan revealing a solitary fibrous tumor located in the right adrenal gland

The patient was referred for endocrinological evaluation. In the physical examination, the patient had normal body weight and blood pressure, without symptoms, stigmata of Cushing syndrome, or virilization. A blood test showed basal glycemia of 113 mg/dl, glycated hemoglobin 5%, and no ionic alterations (including sodium, potassium, magnesium, and calcium levels). Adrenal basal hormones, dexamethasone suppression test, sexual hormones, and 24-hour urine metanephrines were in the normal range. An adrenal computer tomography scan (TC) showed bilobar-distributed liver cysts and a 9 x 7.5 cm heterogeneous solid right adrenal mass. The spleen, pancreas, and left adrenal gland were of normal size, contours, and morphology. Kidneys of normal size and morphology and without duct dilatation. There were no mesenteric, retroperitoneal, pelvic, or inguinal nodes of significant size. No collections, pneumoperitoneum, or free intra-abdominal fluid were observed (Figure 2). 

Based on these findings, the clinical case was presented to the Endocrine Tumors Committee of our hospital and it was decided to perform surgical intervention. The patient underwent robotic anterolateral right adrenalectomy surgery with right adrenal vein dissection. The material was sent to the pathology department.

**Figure 3:**
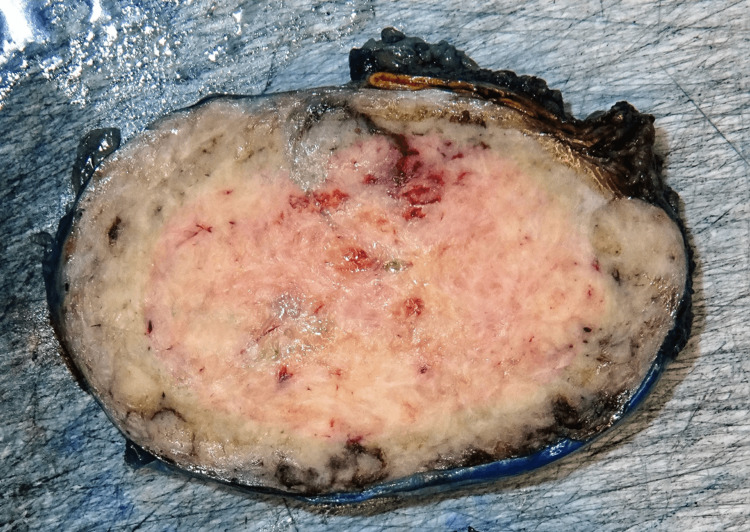
Macroscopic image of the adrenal tumor showing a solid tumor with small hemorrhagic foci and congestive vessels in the peripheral area represented by black color

**Figure 4:**
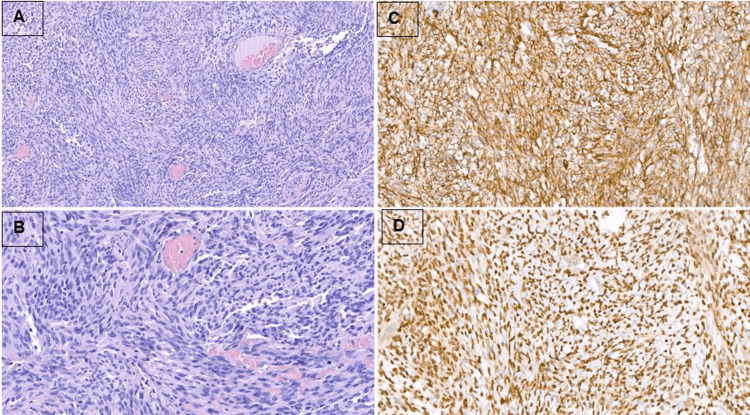
Microscopic images showing a haphazard spindle cell proliferation admixed with branching blood vessels (A - Hematoxilin & Eosin, 200x; B - Hematoxilin & Eosin, 400x). The neoplastic cells are diffusely positive for CD34 (C - 400x) and STAT6 (D - 400x)

Regarding histopathological findings, gross description exposed a 9.5 cm tumor with hemorrhagic areas in intimate contact with the adrenal gland (Figure 3). Microscopic pathological analysis showed a haphazardly arranged spindle cell proliferation mixed with prominent branching blood vessels. Tumor necrosis was not seen, and the mitotic rate was very low (1 mitosis/10 high-power fields). The proliferation was positive for CD34 and STAT6, and negative for desmin, muscle-specific-actin (SMA), S100, SOX10, MUC4, and EMA (Figure 4), and a diagnosis of a solitary fibrous tumor of low risk was performed. Importantly, amplification of the MDM2 gene was ruled out by fluorescence in situ hybridization.

The patient was discharged from the hospital in good condition three days after the surgery. Postoperative laboratory and hormonal findings persisted in the reference range. On the basis of previous literature [4], even though the cases described to date have not recurred, regular follow-up appointments are planned to detect local recurrence or distant metastasis.

## Discussion

Solitary fibrous tumor is an uncommon neoplasm originating from mesothelial mesenchymal cells, constituting less than 2% of all soft tissue masses [5]. While it most frequently presents in the pleura, it can also manifest in various other anatomical sites, including the liver, kidney, retroperitoneum, and prostate, among others. Primary involvement of endocrine organs is rare, and cases of SFTs in proximity to the adrenal gland are often incidentally discovered masses [6]. Actually, as in the case we described, this type of tumor is painless, and symptoms that arise may result from compression of adjacent organs or vascular structures. Notably, in cases documented by Yonly et al. [6] and Kuribayashi et al. [7], symptoms, particularly pain, were reported once the tumor reached approximately 10 cm in size. Another noteworthy consideration is the potential manifestation of paraneoplastic syndromes, such as refractory hypoglycemia due to the insulin-like growth factor release (known as Doege-Potter syndrome) [4,8].

Differential diagnoses include deep benign fibrous histiocytoma, cellular angiofibroma, myofibroblastoma, and low-grade dedifferentiated liposarcoma, among others. Characterizing these masses diagnostically through radiological means remains limited, often revealing a general solid and well-defined nature. In CT imaging, SFTs are frequently depicted as homogenous lesions. However, both benign and malignant lesions can exhibit heterogeneity due to intralesional degeneration and central necrosis [9]. In our case, the CT scan unveiled an adrenal mass displaying a heterogeneous pattern, likely due to the considerable size of the adrenal tumor.

Malignancy is observed in only about 10% of cases, with the majority exhibiting benign behavior. Nevertheless, even benign SFTs have demonstrated instances of locoregional recurrence, emphasizing the necessity for comprehensive risk assessment, surgical intervention involving complete resection, and long-term imaging surveillance. While two established models have facilitated risk assessment [10], the integration of molecular biomarkers holds promise. 

A recent breakthrough has identified the NAB2-STAT6 translocation as almost pathognomonic for SFT, providing valuable diagnostic insight. It is important to note that while STAT6 immunohistochemistry is a sensitive surrogate for detecting STAT6 fusion, it can also exhibit positivity in other entities, such as dedifferentiated liposarcoma, a more commonly occurring tumor in the retroperitoneum. Therefore, the inclusion of MDM2 amplification analysis is imperative in this context to differentiate from dedifferentiated liposarcoma [3,4,11].

Despite not being a specific marker, CD34 is expressed highly in SFTs and is absent in around 5% to 10% of SFTs, mostly in dedifferentiated and malignant instances [1]. As expected, tumor-specific markers of cancers of the nervous system, smooth muscle, fat and melanocytic cells, such as S100, desmin, SMA, and SOX10, are non-reactive with SFT tumor cells, as well as MUC4 and EMA, which are often detected in low-grade fibromyxoid sarcoma.

## Conclusions

Solitary fibrous tumor of the adrenal gland is a rare condition with limited reported cases in the existing literature. Presented here is the case of a woman who exhibited a classical presentation of SFT, with the tumor incidentally detected during imaging studies. This represents the first case diagnosed at our hospital and the initial documented instance in our country. Following surgical intervention, long-term follow-up remains crucial due to documented instances of SFT recurrence over time. Advances in understanding the mutations implicated in SFT carcinogenesis and prognosis could potentially lead to innovative chemotherapeutic protocols.

## References

[1] Rabie A, Hasan A, Mohammed Y, Abdelmaksoud A, Rabaan AA (2022). Recurrent malignant solitary fibrous tumor of the scalp: a case report and literature review. J Pathol Transl Med.

[2] Park SB, Park YS, Kim JK, Kim MH, Oh YT, Kim KA, Cho KS (2011). Solitary fibrous tumor of the genitourinary tract. AJR Am J Roentgenol.

[3] Ambardjieva M, Saidi S, Jovanovic R, Janculev J, Stankov V, Trifunovski A, Popov Z (2021). Solitary fibrous tumor of adrenal gland and review of the literature. Pril (Makedon Akad Nauk Umet Odd Med Nauki).

[4] Jha S, Mohanty SK, Sampat NY (2022). Solitary fibrous tumor of the adrenal gland. Am J Clin Pathol.

[5] Davanzo B, Emerson RE, Lisy M, Koniaris LG, Kays JK (2018). Solitary fibrous tumor. Transl Gastroenterol Hepatol.

[6] Yonli DS, Chakroun M, Mokadem S, Saadi A, Rammeh S, Chebil M (2019). Adrenal solitary fibrous tumor: a case report. Urol Case Rep.

[7] Kuribayashi S, Hatano K, Tsuji H (2019). Solitary fibrous tumor mimicking adrenal tumor concomitant with contralateral adrenal pheochromocytoma: a case report of surgical resection after long-term observation. Int J Surg Case Rep.

[8] Campista-Jácquez JD, Romero-Talamás HR (2021). Solitary fibrous tumor of the left adrenal gland associated with Doege-Potter syndrome. Case report. Cir Cir.

[9] Badawy M, Nada A, Crim J (2022). Solitary fibrous tumors: clinical and imaging features from head to toe. Eur J Radiol.

[10] Pasquali S, Gronchi A, Strauss D (2016). Resectable extra-pleural and extra-meningeal solitary fibrous tumours: a multi-centre prognostic study. Eur J Surg Oncol.

[11] Hasegawa T, Matsuno Y, Shimoda T, Hasegawa F, Sano T, Hirohashi S (1999). Extrathoracic solitary fibrous tumors: their histological variability and potentially aggressive behavior. Hum Pathol.

